# Purification and biological analysis of antimicrobial compound produced by an endophytic *Streptomyces* sp.

**DOI:** 10.1038/s41598-023-41296-x

**Published:** 2023-09-14

**Authors:** Sapna Devi, Manish Sharma, Rajesh Kumari Manhas

**Affiliations:** Department of Microbiology, Guru Nanak Dev University, Amritsar, Punjab 143005 India

**Keywords:** Biotechnology, Microbiology

## Abstract

Fungal phytopathogens and drug-resistant bacteria are two significant challenges in agriculture and public health, respectively. As a result, new sources of antimicrobial compounds are urgently needed. Taking into consideration these aspects, the present study was carried out to explore the antimicrobial activity of *Streptomyces* sp. SP5 against drug-resistant bacteria, especially methicillin resistant *Staphylococcus aureus* (MRSA), vancomycin resistant *Enterococcus * and fungal phytopathogens. MRSA and VRE are both types of antibiotic-resistant bacteria that pose significant challenges to public health. In vitro analysis of the metabolites of *Streptomyces* sp. SP5 exhibited broad-spectrum antimicrobial activity against drug-resistant bacteria and phytopathogenic fungi. Further chemical investigation of the diethyl ether extract led to the isolation and purification of an antimicrobial compound. The structure of the purified compound was elucidated by performing detailed spectroscopic analysis including MS, IR, and NMR. The compound was identified as plicacetin. Plicacetin is a nucleoside antibiotic that has been reported for antibacterial activity against Gram-positive bacterium *Mycobacterium tuberculosis*. According to our knowledge, the present study is the first to demonstrate the antimicrobial properties of plicacetin against *Fusarium oxysporum, Alternaria brassicicola, Fusarium solani*, VRE and *Bacillus subtilis.* The outcome of the current study endorses that compound produced by *Streptomyces* sp. SP5 can be used as an antimicrobial agent against fungal phytopathogens and drug-resistant bacteria.

## Introduction

In agriculture sector emergence of fungal phytopathogens is a major threat for global food security, and more than 20% of the major crop diseases are caused by these pathogens every year^1,2^. The increased resistance of pathogens towards agrochemicals and their effect on a variety of non-target organisms make them unsuitable for long-term management^3,4^. On the other hand, multidrug-resistant bacteria, especially vancomycin-resistant enterococci (VRE) and methicillin resistant *Staphylococcus aureus* (MRSA) have appeared as one of the most serious health threats today, causing severe nosocomial infections in hospitalized patients^5,6^. Recently, the rapid growth of antimicrobial resistance (AMR) has become an extremely serious problem for human health globally and has sparked concern. Microorganisms have developed tolerance to commonly used bioactive secondary metabolites, necessitating the development of new antimicrobial molecules to combat these pathogens^3,4^. The researchers are focusing their efforts on developing novel, potent, long-lasting, and broad-spectrum antimicrobial agents from a variety of sources^7^. Secondary metabolites of microbial origin have the potential to be developed as effective antimicrobial compounds to fight against AMR (David et al. 2015). Among microbial sources, *Streptomyces*, a largest known genus of actinobacteria is the most essential source of antibiotics, as well as one of the most complex bacteria, as it grows as a network of filaments from which aerial branches bearing chains of spores emerge^8^. *Streptomyces* are abundant sources of bioactive natural products with biological activities, which are widely used in the pharmaceutical and agricultural industries. *Streptomyces* spp. play a key role in the development of antifungals, antivirals, antitumoral, and antihypertensive medicines, although just the surface has been scratched thus far^9–13^. Therefore, keeping in mind the continuous demand for potent strains and bioactive compounds, and inspired by the outstanding agricultural and pharmaceutical importance of *Streptomyces* spp., present study was carried out with the objective to purify active compound from *Streptomyces* sp. SP5 possessing antimicrobial activity against fungal phytopathogens and bacterial pathogens.

## Results

### Extraction, separation and bioautography of antimicrobial compound

**Figure 1:**
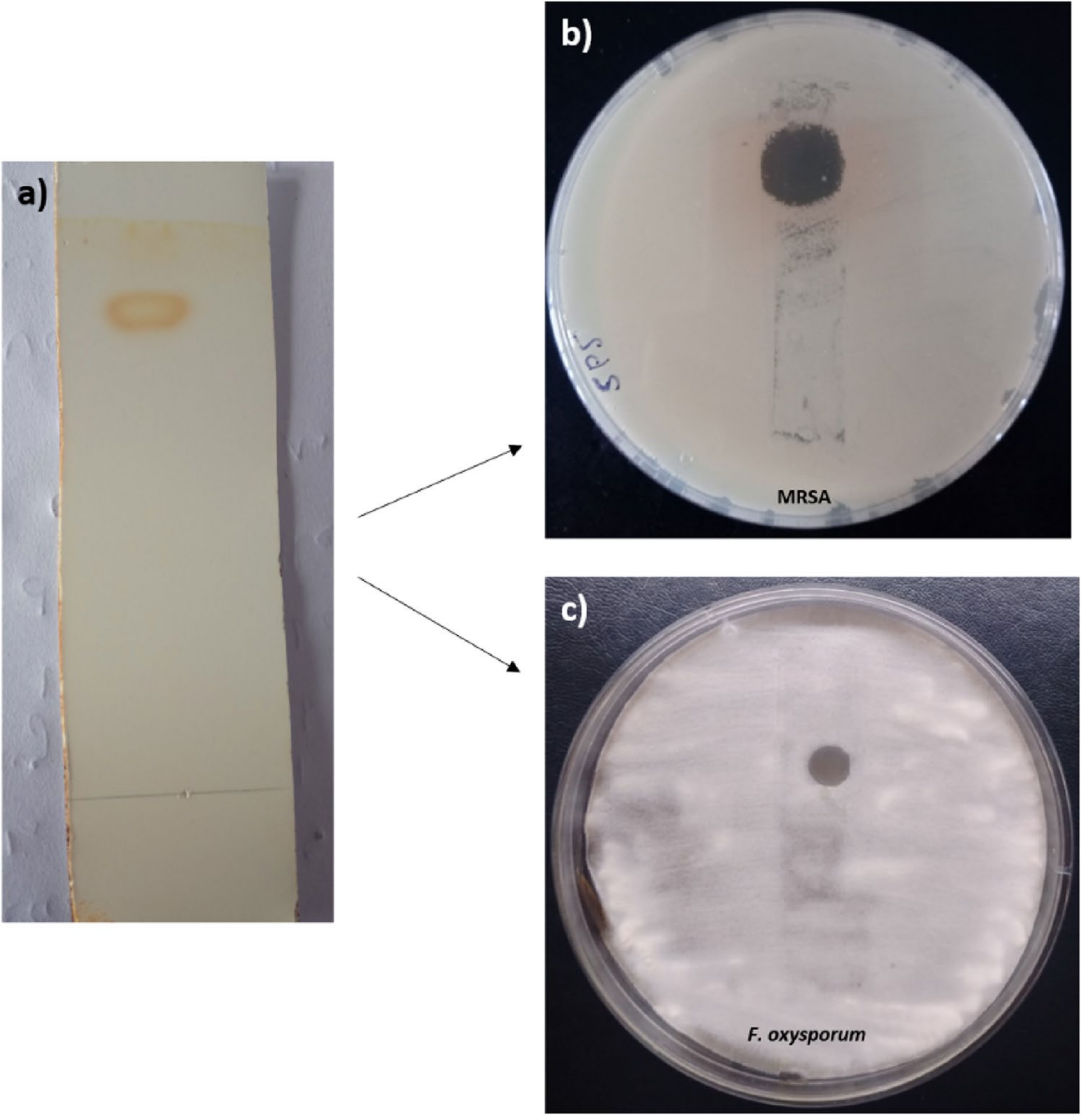
(**a**) Thin layer chromatography of *Streptomyces* sp. SP5 Et_2_ O extract, (**b**) Bioautography of *Streptomyces* sp. SP5 Et_2_ O extract against MRSA, (**c**) Bioautography of *Streptomyces* sp. SP5 Et_2_ O extract against *F. oxysporum.*

Diethyl ether was used to extract antimicrobial metabolites from the culture supernatant because it provided the best recovery of metabolites in terms of inhibition zone. Diethyl ether (Et_2_O) extract showed pronounced activity against *A. brassicicola*, *F. oxysporum,* and *F. solani* (15–20 mm) and Gram-positive bacteria MRSA, VRE, and *B. subtilis* (15–22 mm) (Fig. X7). Thin-layer chromatography of Et_2_O extract followed by bioautography against MRSA and *F. oxysporum* (Fig.  1b,c; X8) revealed the presence of one active compound with an Rf value of 0.9 (Fig.  1a).

### Purification of antimicrobial compound from *Streptomyces* sp. SP5

**Figure 2:**
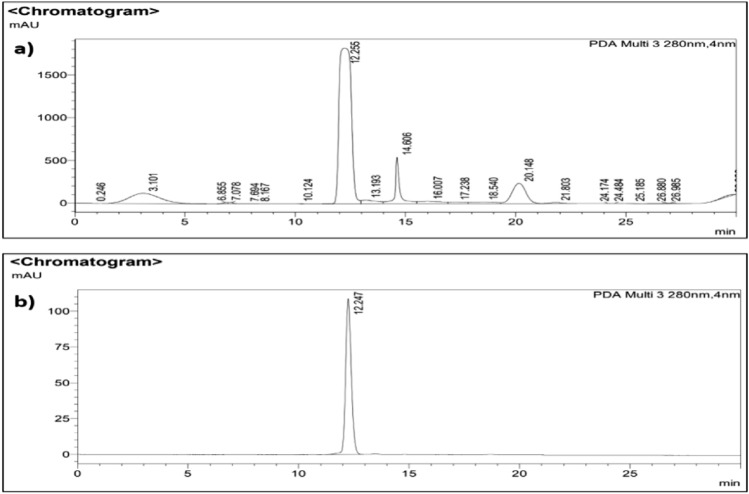
HPLC chromatogram of Et_2_ O extract and SP5P purified compound from *Streptomyces* sp. SP5: (**a**) fractions (32–40), (**b**) Purified compound** (**SP5P).

For the purification of the antimicrobial compound, Et_2_O extract was subjected to silica gel column chromatography. Antimicrobial activity against MRSA and *F. oxysporum* was found in eight fractions (32–40 fractions) eluted with chloroform: ethyl acetate (60:40, v/v). These active fractions were pooled and finally subjected to semi-preparative HPLC. Four prominent peaks with 3.1, 12.25, 14.6 and 20.1 min retention times were found during HPLC analysis using acetonitrile: water (95:5), (Fig. 2a). The antimicrobial activity was detected in the peak with a retention time of 12.25 min. This active peak was further chromatographed using acetonitrile: water (95:5). A single peak with a retention time of 12.247 min was obtained, confirming the compound’s purity (Fig.  2b). The purified compound was named SP5P.

### Structure elucidation of the compound purified from *Streptomyces* sp. SP5

The purified compound, SP5P (25 mg) with a retention time of 12.247 min was characterized as plicacetin using spectroscopic techniques viz*.* LC–MS, UV–Visible, ^1^H-NMR, and FT-IR. It was a white crystalline needle shaped compound, soluble in chloroform, methanol, diethyl ether, DMSO and acetonitrile, but sparingly soluble in water. The molecular formula, determined as C_25_H_36_N_5_O_7_ for ion peak at m/z 518.3124^(M+H)^ (Fig. X1) (C: 12.01, H: 1.00794, N: 14.0067, O: 15.9994 g/mol), and UV_λmax_ 295 (225) nm^20,22^ and ^1^H NMR^41,42^ (Fig. X2; Table 1), were identical to plicacetin. FT-IR (KBr): v_max_ (cm^−1^) showed the presence of different functional groups viz*.* hydroxyl (3332.2), alkyne (2117.1), carbonyl (1736.9), alkene (1513.3), and aromatic rings (1028.5 and 872.2). At about 2929.7, 1371.1 and 1095.8–1282.2 cm^−1^ band occurs due to C–H, C–N and C–O–C linkages, respectively (Fig. X3). The structure of compound SP5P is shown in (Fig. X4).

**Table 1:** ^1^H NMR spectrum of compound SP5P.

Moiety	No.	Chemical shift (δH)	Chemical Shift (δH) Aryal et al.^41^	Chemical shift (δH) Aksoy et al.^42^
Cytosine	5	7.62 d	7.59 d	7.34 d
	6	8.72 s	8.11 d	8.16 d
Side chain	10	7.74 d	7.76 d	8.07 d
	11	6.79 d	6.69 d	7.79 d
	13	6.79 d	6.69 d	7.79 d
	14	7.76 d	7.76 d	8.07 d
Amicetose	1′	5.81 d	5.76 d	5.73 d
	2′	1.75 m, 2.04 m	1.65 m, 2.15 m	1.73 m, 2.07 m
	3′	1.75 m, 3.38d	1.65 m, 2.38 m	1.5 m, 2.29 d
	4′	3.23 m	3.41 m	3.36 m
	5′	3.51 m	3.75 m	3.65 m
	6′	1.36 d	1.36 d	1.16 d
Amosamine	1″	4.97 d	4.92 d	4.81 d
	2″	3.42 m	3.41 m	3.27 dd
	3″	4.31 t	3.87 t	3.77 t
	4″	1.25 t	2.13 t	2.38 s
	5″	4.11 m	3.83 m	3.63 m
	6″	1.28 d	1.24 d	1.27 d
	7″	2.36 s	2.47 s	
	8″	2.36 s	2.47 s	

### Antimicrobial activity and MIC values of purified compound

**Figure 3:**
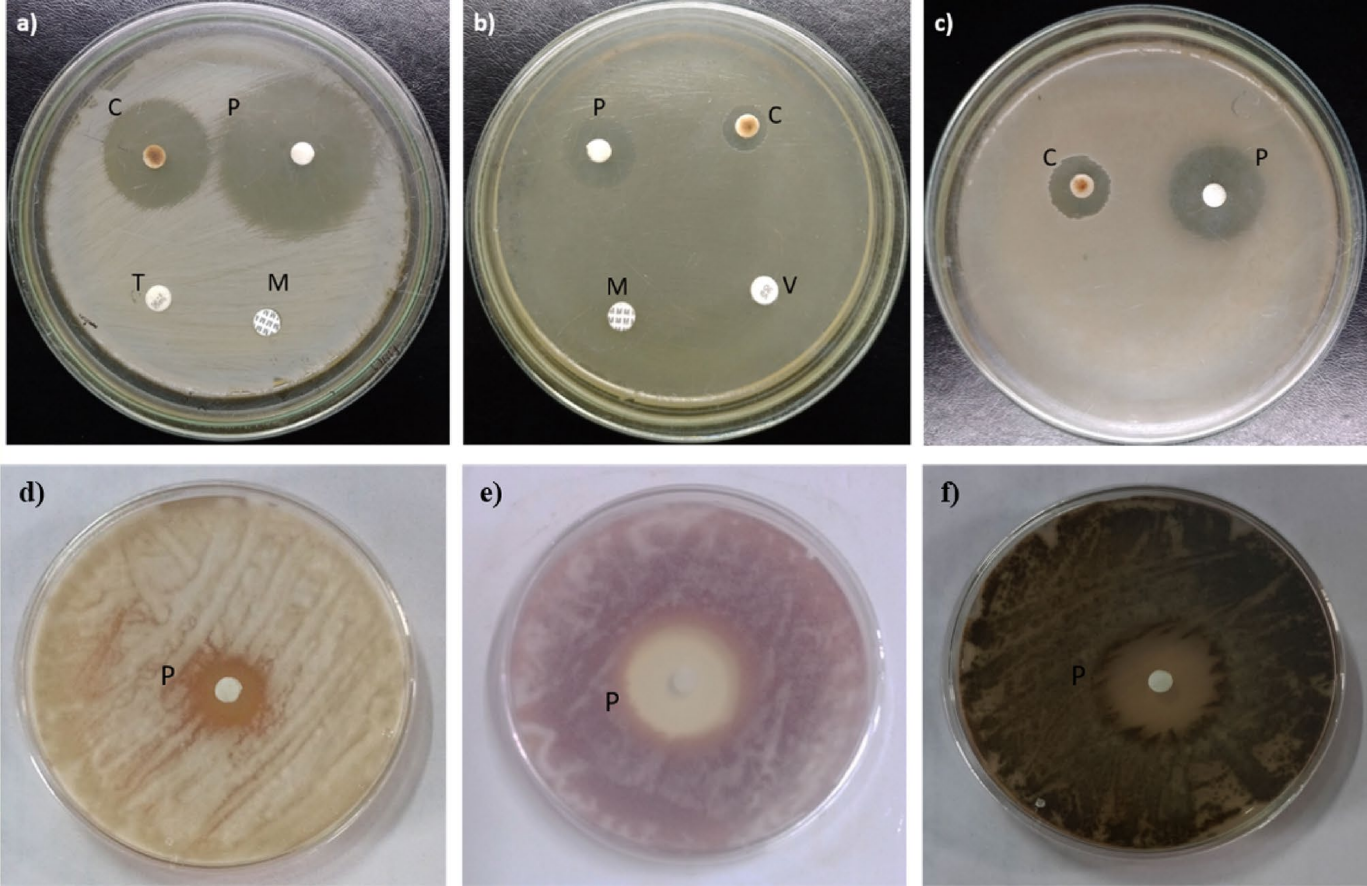
Antimicrobial activity of *Streptomyces* sp. SP5 compounds against: (**a**) MRSA, (**b**) VRE, (**c**) *B. subtilis,* (**d**) *F. solani*, (**e**) *F. oxysporum*, (**f**) *A. brassicicola*. C: Crude Et_2_ O extract, P: Purified compound, T: Teicoplanin (30 µg/disc), M: Methicillin (10 µg/disc), V: Vancomycin (30 µg/disc).

The purified compound exhibited potent antimicrobial activity against Gram positive bacteria viz. MRSA, VRE, *B. subtilis* and fungal phytopathogens *F. oxysporum, A. brassicicola*, *F. solani* (Fig. 3). The compound was more effective against Gram positive bacteria as compared to fungal phytopathogens. Among bacteria plicacetin was found to be more effective against MRSA (MIC:3.8 μg/ml) and *B. subtilis* (MIC:3.8 μg/ml) as compared to VRE (MIC:15.6 μg/ml). MIC values of plicacetin against fungal phytopathogens were determined to be 3.8 μg/ml for *F. oxysporum and A. brassicicola,* and 15.6 μg/ml for *F. solani*.

### Scanning electron microscope (SEM) studies of microbial cells treated with plicacetin

**Figure 4:**
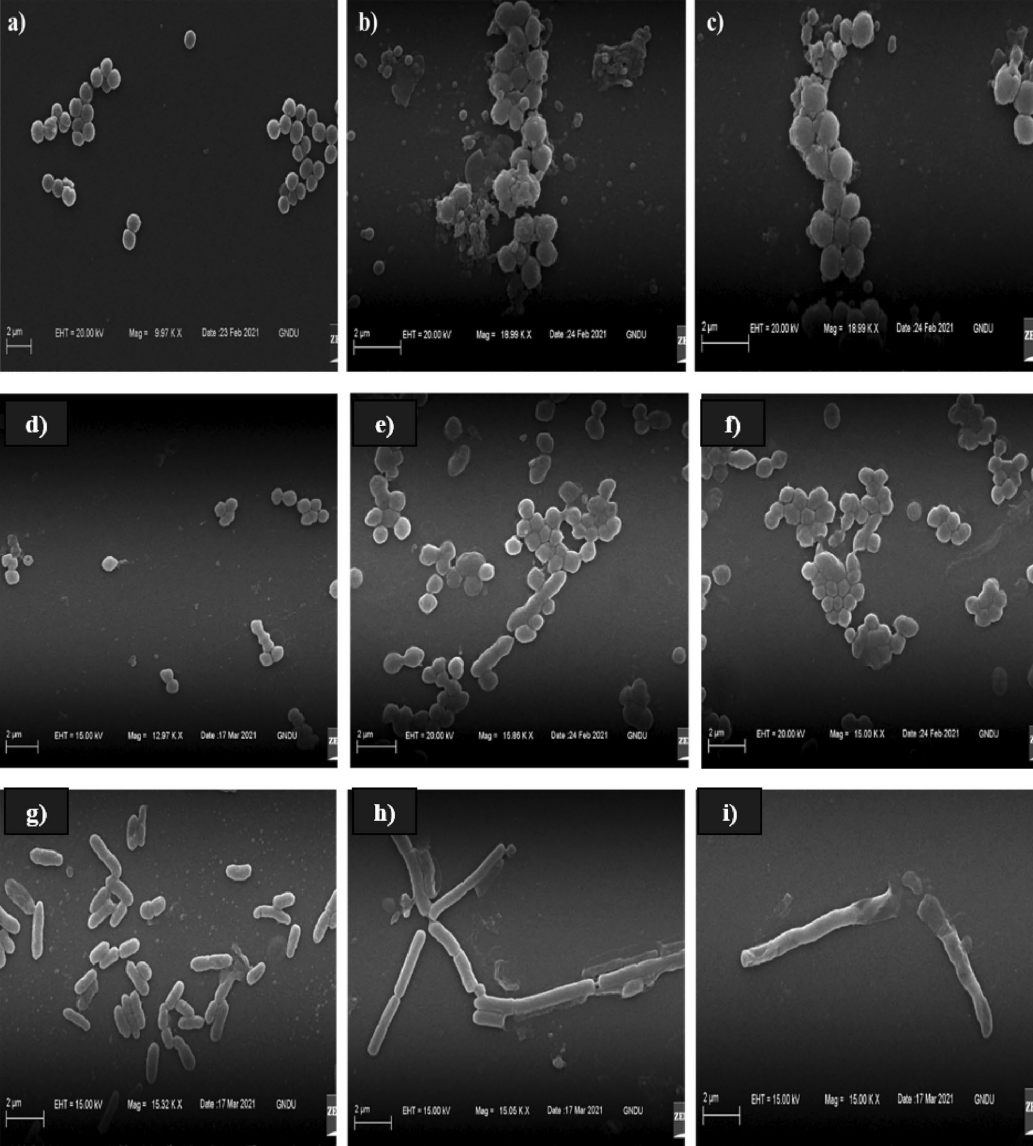
The Scanning electron micrographs showing the effect of plicacetin on MRSA, VRE and *B. subtilis*: Control (untreated cells); (**a**) MRSA, (**d**) VRE, (**g**) *Bacillus subtilis*; Cells treated with plicacetin; (**b**) MRSA treated with plicacetin, (**e**) VRE treated with plicacetin, (**h**) *B. subtilis* treated with plicacetin; Positive control; (**c**) MRSA treated with Vancomycin; (**f**) VRE treated with teicoplanin i) *Bacillus subtills* treated with Gentamicin.

Plicacetin induced a variety of cell deformities and intracellular material leakage, according to SEM research. The electron micrograph of control MRSA cells revealed smooth, undamaged spherical cells that were present individually or in clusters with a distinct boundary between the cells (Fig. 4a). The plicacetin-treated MRSA cells, on the other hand, displayed a variable cell deformity (Fig. 4b). The treated cells clumped together and formed an adhered floc surrounded by oozed-out cellular material, indicating ruptured cell walls and membranes, as well as lysed and fully burst cells. Cells treated with vancomycin were seen as twisted and clumped entities, surrounded by the intracellular material produced by MRSA cells losing their cytoplasm (Fig. 4c). VRE untreated (control) cells appeared smooth-surfaced and spherical to elongate in shape (Fig. 4d). However, VRE cells treated with plicacetin had a skewed cell morphology (Fig. 4e). The treated cells clumped together in most cases, with a small depression on the surface. The cells had a rough, wrinkled surface and could be seen as clusters of interconnected cells. Along with the deformed and clumped cells, the micrographs revealed some heavy deposition of flocculated material on the cell surface. Cells were clumped and flattened, surrounded by the oozed out cellular material, similar to that caused by teicoplanin (Fig. 4f).

Similarly, *B. subtilis* normal (untreated) cells were rod-shaped, smooth-surfaced, and had an intact cell wall, exhibiting a distinct morphology (Fig. 4g). Cells exposed to plicacetin, on the other hand, showed visible disruptions and anomalies in cellular morphology, such as irregular and rough surfaces. The cells seemed to be elongated and clumped together (Fig. 4h). Gentamicin had a similar effect, causing extreme cell deformities in the form of elongated, irregular, and clustered cells, which were somewhat different from normal cell morphology (Fig. 4i). Plicacetin was reported for its disruptive effect on test bacteria similar to that in case of standard antibiotics which was evidenced by deformed cellular morphology and oozed-out cellular content, that might be due to increased cell permeability.

### Safety evaluation of the Et_2_O extract and purified compound (SP5P)

#### Ames assay: evaluation of mutagenicity

*Streptomyces* sp. SP5 metabolites (Et_2_O extract and SP5P compound) were found to be non-mutagenic against TA98 and TA100 (S. Typhimurium strains). In the absence of mutagen, the number of spontaneous revertant colonies was found to be 142 ± 1.4 and 168.3 ± 0.6 for TA98, and 354.3 ± 2.2 and 232.3 ± 1.7 for TA100. Colonies in the presence of SP5 metabolites were comparable to spontaneous revertant colonies which revealed nonmutagenic nature of the SP5 metabolites.

**Figure 5:**
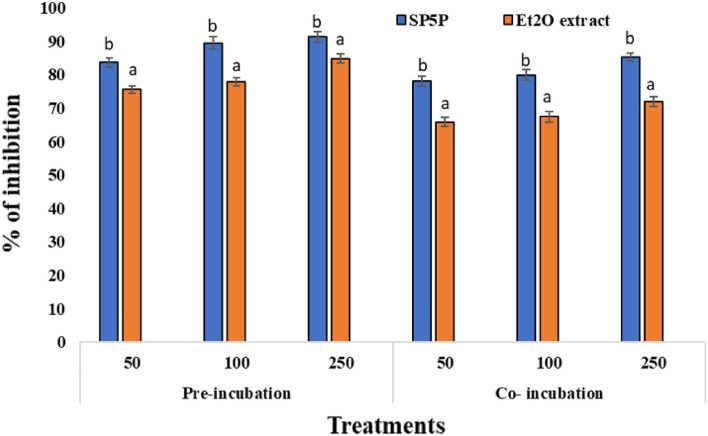
Comparative analysis of the antimutagenic activity of SP5P and Et_2_O extract against *S.* Typhimurium TA98 strain. Different letters a and b on graphs represent the significant difference (Tukey’s Test *p* ≤ 0.05), whereas the same letters represent no significant difference.

**Figure 6:**
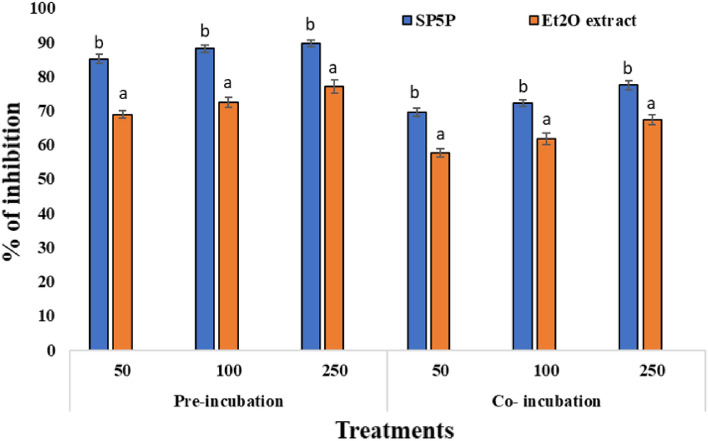
Comparative analysis of antimutagenic activity of SP5P and Et_2_O extract against *S.* Typhimurium TA100 strain. Different letters a and b on graphs represent significant differences (Tukey’s Test *p* ≤ 0.05), whereas the same letters represent no significant difference.

However, metabolites from *Streptomyces* sp. SP5 showed antimutagenic activity against S. Typhimurium strains. In the three experiments, carried out independently to check the antimutagenic activity of SP5 metabolites, the number of revertant colonies in the positive control (in the presence of direct-acting mutagens i.e., NPD) was found to be 780.3 ± 1.8 and 982 ± 1.5 for TA98, and 1055 ± 1.8 and 1373 ± 2.5 for TA 100 (Table S1, S2). However, in the antimutagenicity assay SP5 metabolites showed significant antimutagenic response against NPD (in the case of TA98) and sodium azide (TA100). In the two modes of treatments, metabolites displayed more antimutagenic activity in pre-incubation treatment and were found to be more effective against TA98 than TA100 (Fig. 5, 6).

The antimutagenic response of compound SP5P purified from *Streptomyces* sp. SP5 was more pronounced as compared to Et_2_O extract in terms of percentage inhibition (Table S1). Et_2_O extract exhibited significant antimutagenicity against both mutagens with inhibition of 71.95% and 67.40% against NPD (TA98) and sodium azide (TA100), respectively in co-incubation, and inhibition of 84.85 and 76.99%, respectively was observed during pre-incubation (250 µg/100 µl concentration) (Table S1). Compound SP5P, showed more effective activity as compared to Et_2_O extract against both mutagens with inhibition of 91.32% and 89.63% against NPD and sodium azide, respectively in pre-incubation, and inhibition of 85.22 and 77.43%, respectively was observed during co-incubation (at 250 µg/100 µl concentration) (Table S2).

#### DNA nicking assay: to check the protective effect of *Streptomyces *sp. SP5 against oxidative damage induced by H_2_O_2_

Et_2_O extract and SP5P compound demonstrated a protective effect on the supercoiled pBR322 DNA from the destructive action of hydroxyl radicals released by Fenton’s reagent. The free OH^−^ (hydroxyl) radicals converted supercoiled pBR322 DNA to Form II (single-stranded nicked) and Form III (linear). However, SP5 metabolites minimized the effect of hydroxyl radicals by protecting the DNA Form I (supercoiled) from breaking into DNA Forms II and III (Table S3). Rutin, utilized as a positive control in the reaction maintained the supercoiled pBR322 DNA (Form I) integrity.

**Figure 7:**
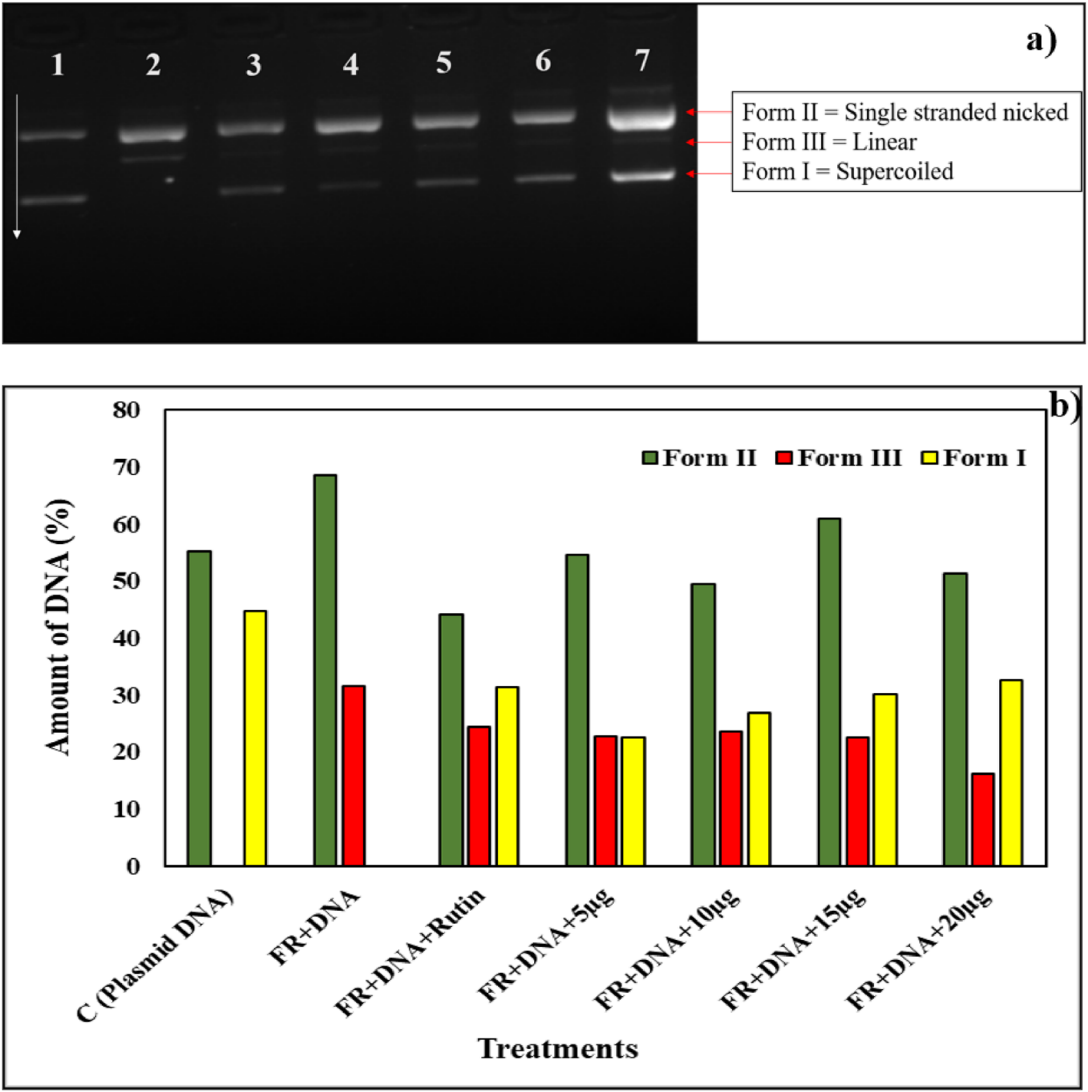
(**a**) DNA protective potential of Et_2_O extract, Well 1 (negative control): only plasmid DNA; Well 2: Fenton’s reagent; Well 3: Positive control (Rutin, 10 μg); Wells 4–7: Fenton’s reagent + different concentrations of Et_2_O extract (5, 10, 15 and 20 μg/well); Supercoiled (Form I); Single strand nicked DNA (Form II); Linear (Form III); (**b**) Densitometric analysis of pBR322 plasmid DNA treated with Et_2_O extract in the presence of Fenton’s reagent (original gel are presented in Supplementary Fig. X5).

**Figure 8:**
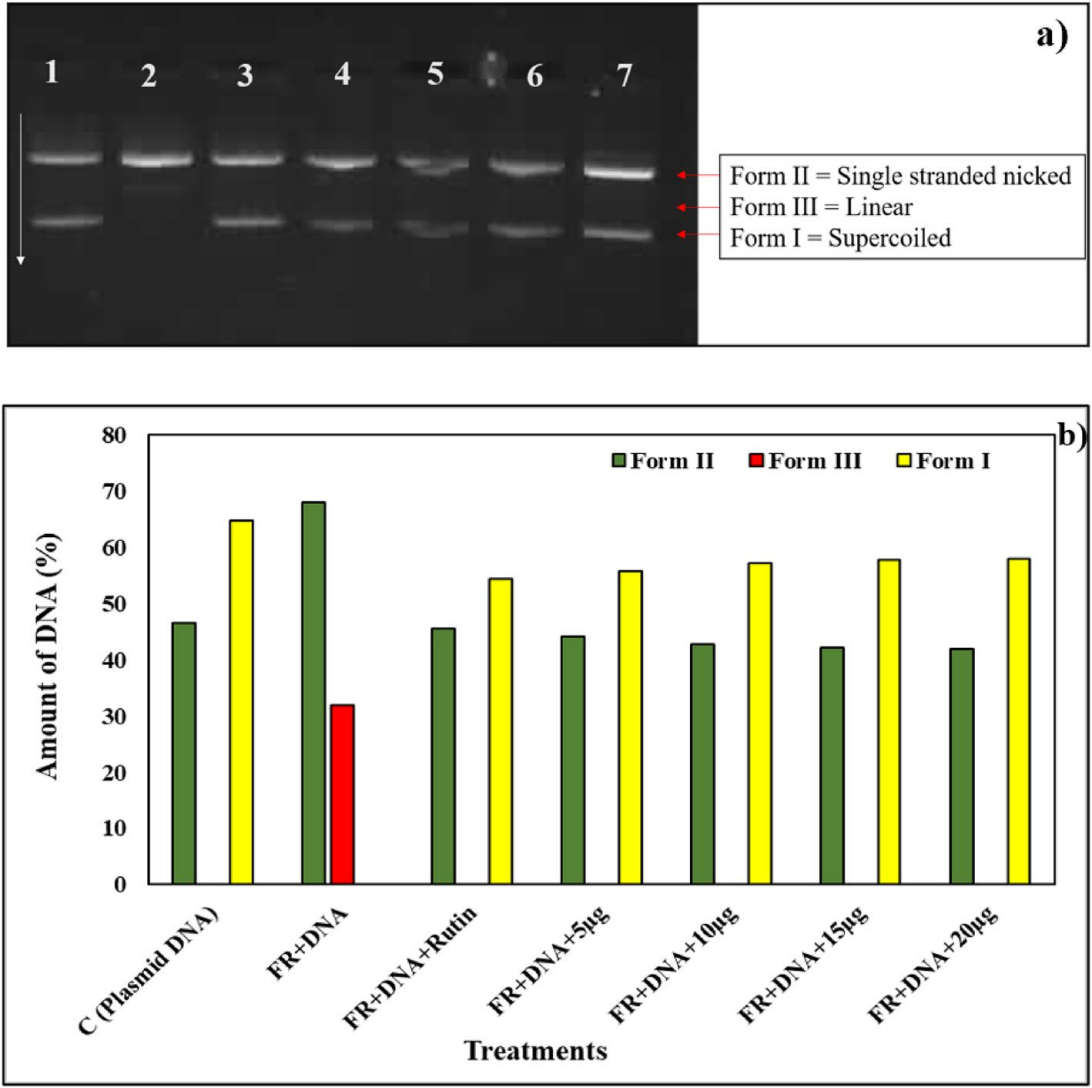
(**a**) DNA protective potential of SP5P compound purified from SP5 against reactive oxygen species generated by Fenton’s reagent. Well 1 (negative control): only plasmid DNA; Well 2: Fenton’s reagent; Well 3: Positive control (rutin, 10 μg); Wells 4–7: Fenton’s reagent + different concentrations of SP5P compound (5, 10, 15 and 20 μg/well); Supercoiled (Form I); Single strand nicked DNA (Form II); Linear (Form III); (**b**) Densitometric analysis of pBR322 plasmid DNA treated with SP5P compound in the presence of Fenton’s reagent (original gel are presented in Supplementary Fig. X6).

The amount of DNA present in three forms was determined by the densitometric analysis. The amount of supercoiled pBR322 DNA (Form I) in the presence of Et_2_O extract and Fenton’s reagent was 22.587% (at 5 µg), 26.891% (at 10 µg), 30.268 (at 15 µg), and 32.619 (at 20 µg) which showed that with increasing concentration of Et_2_O extract the supercoiled DNA protection increased, showing higher intensity (Fig. 7a,b). The plasmid DNA treated with Fenton’s reagent in the presence of SP5P compound resulted in an increase of 55.833%, 55.198%, 57.873%, and 57.996% in supercoiled DNA at 5 µg, 10 µg, 15 µg, 20 µg of compound SP5P, respectively. In the presence of the SP5P compound Form III was not formed which indicated that the SP5P provided high protection from oxidative DNA damage by the Fenton reagent (Fig. 8a,b).

#### *In vitro* cytotoxicity using MTT assay

**Figure 9:**
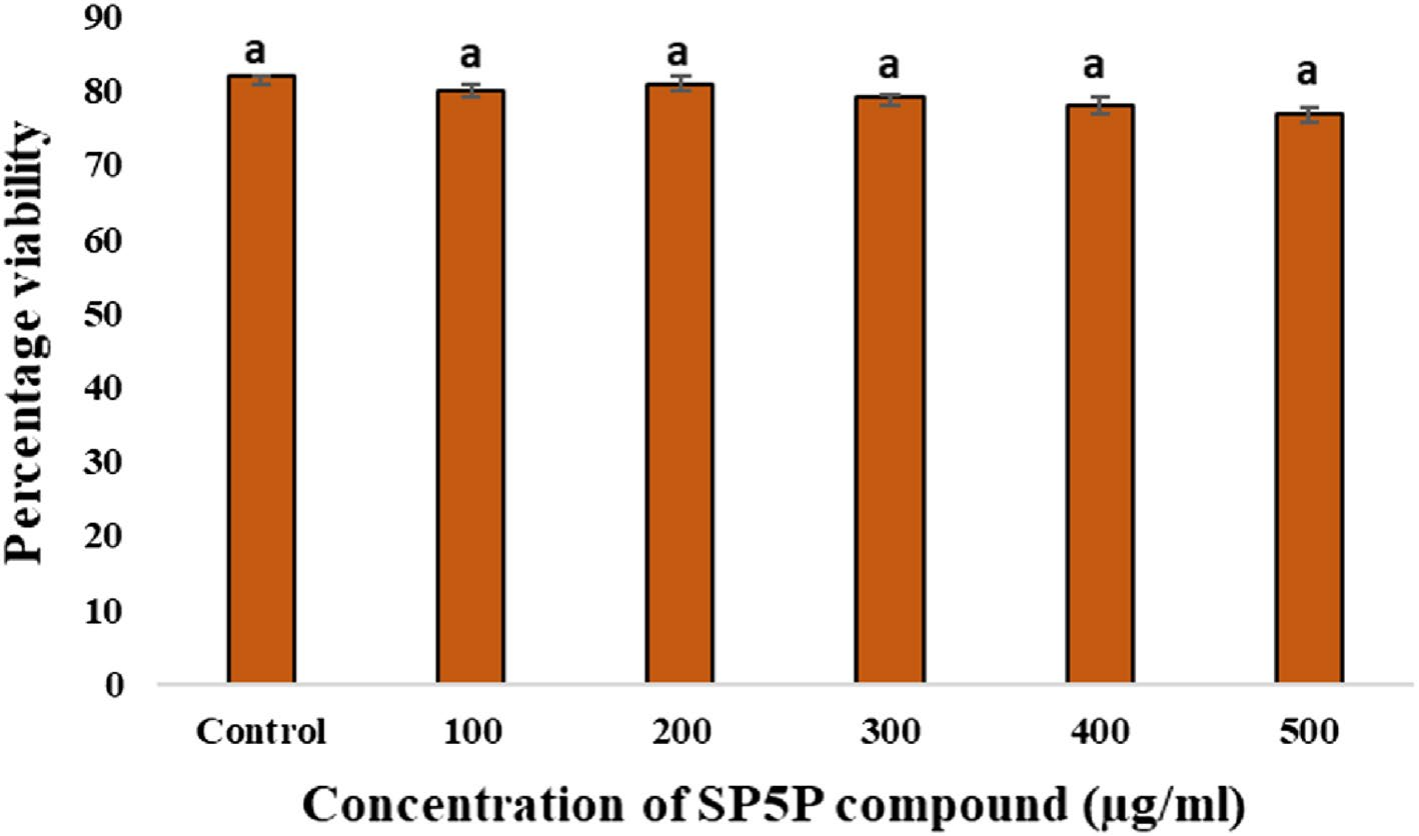
Effect of SP5P compound on HEK-293T normal cell line. The viability of control cells (without compound) was defined as 100%. Data shown are mean ± SE (*n* = 3), error bars with same letter “a” denotes *p* ≥ 0.05 between control cells and treated cells i.e. statistically insignificant difference.

MTT assay displayed an insignificant cytotoxic effect of the SP5P compound isolated from *Streptomyces* sp. SP5 on HEK-293T normal cell line, showing viability ranging from 80.09 ± 0.87 to 76.87 ± 0.65% at the different tested concentrations (Fig. 9). No significant difference (*p* ≥ 0.05) was observed between the control and increasing concentrations (100–500 µg/mL) of the SP5P compound.

## Discussion

Numerous harmful fungi and bacteria continue to evolve novel ways to adapt and withstand the deadly or biostatic effects of antimicrobials, necessitating the development of novel antibacterial and antifungal compounds to tackle these pathogens^18^. Filamentous soil bacteria belonging to the genus *Streptomyces* are abundant sources of bioactive natural compounds with biological activities that are widely employed as medicines and agrochemicals^19^. In the present investigation a white crystalline  antimicrobial compound was  purified from the culture supernatant of *Streptomyces* sp. SP5 isolated from *Citrus jambhiri* leaves, and identified as plicacetin on the basis of LC-MS, FTIR and NMR spectral data.

The physical and spectral data of plicacetin were comparable to the published values that further confirmed these findings. Evans and Weare^20^ also isolated plicacetin in white crystal form streptomycete. Plicacetin belongs to the nucleoside antibiotics group amicetin^21^ and was first time reported by Haskell et al.^22^ from the culture filtrate of *Streptomyces plicatus*. Amicetin, the first antibiotic in the amicetin family, was identified in the fermentation broth of *Streptomyces*, *Arthrobacter*, and *Nocardia* species in the early 1950s and was shown to have antibacterial and antiviral properties^23–25^. The purified plicacetin showed potent antimicrobial activity against Gram-positive bacterial and fungal phytopathogens with inhibition zones ranging from 18-30 mm. It showed MIC values of 3.8 μg/mL against MRSA, *B. subtilis*, *A. brassicicola and F. oxysporum,* and 15.6 μg/mL against VRE and *F. solani.* Plicacetin was described first time for antibacterial activity against *Mycobacterium tuberculosis* only by Haskell et al. in 1958. Later, Bu et al.^26^ isolated plicacetin from *Streptomyces* sp. TPU1236A culture broth showed its MIC value of 32 μg/mL against *M. tuberculosis.* However, in the current study, we have demonstrated for the first time the antimicrobial activity of plicacetin, purified from *Streptomyces* sp. SP5, against drug-resistant bacteria MRSA, VRE, and fungal phytopathogens. It is not surprising that plicacetin exhibited antimicrobial activity because nucleoside antibiotics have a wide range of biological functions, they act on nucleosides and nucleotides that play important roles in fundamental cellular metabolism, such as energy donors, secondary messengers, metabolite carriers, and cofactors for numerous enzymes^27–29^.

Scanning electron microscopy (SEM) was used to view the morphological changes that occurred in the cells as a result of exposure to the plicacetin, which could provide insight into its potential mechanism of action. Most of antibacterial agents act on the cell wall and cell membrane^27^. According to Hartmann et al.^31^, gramicidin S and PGLa cause severe membrane damage, resulting in an immediate bactericidal effect. Aruldass et al.^32^ also discovered that the antibacterial agent violacein inhibits bacterial growth by disrupting membrane integrity, morphological changes, and cell membrane rupture. During morphological studies, plicacetin also showed leaked cytoplasmic material, which could be due to increased cell membrane permeability, disintegrated cell wall, and anomalies in cellular morphology.

It is necessary to determine the toxicity of active metabolites in order to market them as safe antimicrobial drugs, biofungicides, and biopesticides in the clinical and agricultural fields. Ames mutagenicity and DNA nicking assays are the most accessible and reliable procedures for determining the biosafety of a compound. The Ames mutagenicity assay, also known as the reverse mutation assay, was invented by Ames^33^ and is a widely used method in experimental studies to detect the mutagenesis of compounds. Salmonella Typhimurium strains TA98 and TA100 with a mutation in the histidine gene (his- mutant) are used in this assay to determine whether the tested compound produces frameshift mutations and base-pair substitution in DNA (GC base pairs). *Streptomyces* sp. SP5 Et_2_O extract and SP5P purified compound were revealed to be non-mutagenic against S*.* Typhimurium. Instead, in the presence of mutagens, Et_2_O extract and SP5P compound showed significant antimutagenic activity i.e. 84.85%; 89.63% and91.32%; 89.63% against 4-Nitro-o-phenylenediamine and sodium azide, respectively for TA98 and TA100 at 250 µg/100 µl. The antimutagenic action was found to be dose-dependent, which is consistent with previous results that revealed a concentration-dependent antimutagenic response of several natural extracts against *Salmonella* strains^34,35^. Kaur et al.^36^ also reported the antimutagenic potential of a new antifungal compound SH2 produced by *S. hydrogenans* strain DH16 against S. Typhimurium strains, with 70% suppression at 250 g/100 µl. Similarly, Sharma and Manhas^30^ demonstrated an inhibition of 47.87–88.11% and 41.10–88.53% for TA98 and TA100 at 250 μg/100 μL by salvianolic acid B purified from *Streptomyces* M4. Further, DNA nicking assay demonstrated the potential of both Et_2_O extract and plicacetin produced by *Streptomyces* sp. SP5 to protect DNA from oxidative stress-induced DNA damage. Et_2_O extract minimized the conversion of pBR322 plasmid DNA Form I to DNA Forms II and III (generated by Fenton’s reagent) by hydrogen abstraction mechanism. In the presence of purified plicacetin, DNA Form III could be attributed to a reduction in the production of free hydroxyl radicals^37–39^. According to Karthik et al*.*^40^ extracts of marine actinobacteria isolates protected the DNA from oxidative stress. Similarly, the DNA protective characteristics of EtOAc extract of *S. cellulosae* strain TES17 were reported by Rani et al.^39^, who found that it boosted the native supercoiled form (Form I) and lowered Form II (single-stranded or double-stranded nicked) and Form III (linear form). The findings of the current study concluded that *Streptomyces* sp. SP5 can be exploited to develop drug leads against rising drug-resistant bacteria and to control fungal phytopathogens.

## Conclusion

The plicacetin produced by *Streptomyces* effectively acts against drug resistant bacteria, especially MRSA and VRE, and fungal phytopathogens *F. oxysporium*, *F. solani* and *A. brassicicola*. The current findings concluded that *Streptomyces* sp. SP5 could be exploited to develop drug leads against rising drug resistant bacteria and fungal phytopathogens.

## Material and methods

### Chemicals and media

The chemicals, media components and the standard antibiotics used in the study were purchased from Hi Media Pvt. Ltd. Mumbai, India and Sigma-Aldrich Corporation, Bangalore, India. All solvents used were of analytical/ HPLC grade and were purchased from SD Fine Chem. Limited India. The pre-coated silica gel F_254_ (plates and powdered form) (Merck, Dermstadt, Germany and S D fine-chem limited) were used for thin layer chromatographic analysis.

### Microbial culture and test organisms

Endophytic *Streptomyces* sp. SP5 (GenBank accession number MW564023) isolated from *Citrus jambhiri* leaves was used in the current study^14^. The streptomycete was maintained on starch casein nitrate agar (SCNA) plates, and spores of isolate were preserved in 20% glycerol at − 20 °C as stock for future use. Different test pathogenic bacteria viz. *Bacillus subtilis* (MTCC 619), *Klebsiella pneumoniae* subsp. *pneumoniae* (MTCC 109), *E. coli* (MTCC 1885), *Staphylococcus epidermidis* (MTCC 435), *Salmonella typhi* (MTCC 733), *Alternaria brassicicola* (MTCC2102), *Colletotrichum acutatum* (MTCC1037), *Alternaria solani* (MTCC2101), *Cladosporium herbarum* (MTCC351) and *Fusarium oxysporum* (MTCC284) were obtained from the Microbial Type Culture Collection (MTCC) and Gene Bank, CSIR-Institute of Microbial Technology (IMTECH), Chandigarh, India. *Fusarium solani* (NFCCI 91) was obtained from the NFCCI (National Fungal Culture Collection of India), Pune, and *Alternaria alternata* (accession number GU004283) and *Fusarium moniliforme* were isolated in the lab (PAU). VRE (resistant to methicillin, vancomycin, imipenem, and clindamycin) and MRSA (resistant to methicillin, teicoplanin, imipenem, and clindamycin) were collected from local hospitals. The bacterial and fungal cultures were maintained at 4 °C on nutrient agar and potato dextrose agar slants, respectively.

### Recovery of antimicrobial metabolites

The antimicrobial metabolite production by *Streptomyces* sp. SP5 was carried out as described by Devi et al.^14^. For the production of antimicrobial metabolites, fermentation was carried out in a production medium (Starch casein nitrate broth, pH 7), inoculated with 2.5% seed culture, at 28 °C under shaking (180 rpm) for 5 days (time at which the maximum antifungal activity was observed). To separate the mycelium, culture broth was centrifuged at 10,000×*g* at 4 °C for 10 min. Diethyl ether solvent was used to extract active metabolites from the culture supernatant.

### Bioautography

Thin layer chromatography (TLC), with ethyl acetate: chloroform (9:1, v/v) solvent system, was used to analyse the antimicrobial compounds present in the di-ethyl ether extract (Et_2_O extract). The chromatogram was observed in iodine vapors and under UV light. To determine the number of antimicrobial compounds, TLC strips were mounted aseptically on the surface of MHA and PDA plates that had already been seeded with the test cultures MRSA and *F. oxysporum,* respectively. For 1 h at 4 °C, the TLC strips on the agar plates were incubated so that the active metabolites could diffuse into the agar. The plates were then incubated for 24–48 h at 37 °C and 28 °C for bacteria and fungi, respectively*.* The presence of inhibition zones, which indicate the number of active compounds in the solvent extract, was observed.

### Purification and characterization of the active compound

Silica gel chromatography was used to purify antimicrobial compounds from di-ethyl ether extract (50 mg). The column (35 × 1.0 cm) was packed with silica gel (60–120 mesh) using chloroform as solvent and eluted step-by-step with 100% chloroform, 90:10, 80:20, 70:30, 60:40, 50:50, 40:60, 30:70, 20:80, 10:90 (v/v) of chloroform: ethyl acetate, 100% ethyl acetate (200 ml each) at a flow rate of 2 ml/min. A total of 88 fractions (25 ml each) were collected and concentrated. All fractions were subjected to antimicrobial activity against MRSA and *F. oxysporum* using the standard Kirby-Bauer disc diffusion method. MHA and PDA plates were swabbed with 100 μl test bacterium (OD equivalent to MacFarland standard 0.5) and fungus (10^6^ spores/ml). Discs (6 mm) loaded with residue obtained from concentrated fractions were placed on the medium plate followed by compound diffusion at 4 °C for 30 min. The plates were incubated at their respective temperatures and the zones of inhibition were measured. Fractions showing antimicrobial activity against MRSA and *F. oxysporum* were pooled and further fractionated using RP-HPLC (reversed-phase high performance liquid chromatography: Shimadzu Micros orb MV, 100 mm × 10 mm ID, 10 μm), at a flow rate of 1 ml/min with a mobile phase of acetonitrile: H_2_O (90:10) in 30 min. A fraction collector connected to an HPLC system was used to collect chromatogram peaks, which were then concentrated and screened for antimicrobial activity.

To deduce the structure of the purified molecule, various spectroscopic techniques were used. Using chloroform as a reference solvent, the UV–Visible spectra were recorded qualitatively on a Shimadzu UV–Visible Spectrophotometer in the range of 200–800 nm. The mass spectrometry (MS) was performed with a Bruker MICROTOF II spectrometer, the Fourier transformation infrared spectroscopy (FT-IR) was recorded in the range 400–4000 cm^−1^ with a Perkin–Elmer Spectrum RX-IFTIR spectrophotometer, and nuclear magnetic resonance (NMR) spectroscopy was performed in chloroform-d [99.8 atom% D, containing 0.1% (v/v) tetramethylsilane (TMS)] at 25 °C on 500 MHz AVANCE III Bruker spectrometer equipped with a 5 mm double-channel solution state probe.

### Antimicrobial activity and minimum inhibitory concentration (MIC) of purified compound

The purified compound was tested for antimicrobial activity against MRSA, VRE, *B. subtilis,* and phytopathogens *F. oxysporum, A. brassicicola*, *F. solani* using the standard Kirby-Bauer disc diffusion method. For determining the MIC of the purified compound against bacterial pathogens VRE, MRSA, and *B. subtilis* (between 0.3 to 0.5 OD at 595 nm), a 96-well microtiter plate dilution assay was used. The purified compound (1.97, 3.95, 7.56, 15.12, 31.25, 62.5, 125 µg/ml) was prepared in water. 100 μl of bacterial culture was mixed with 100 μl of test compound, control blanks contained 100 μl of test compound with 100 μl of nutrient broth, positive control well consisted of 100 μl of bacterial culture and 100 μl of Nutrient broth (NB), negative control contained 200 μl of NB only. The plates were incubated and OD was measured at 595 nm using an ELISA microplate reader (Bio-Rad, Model 680XR).

A fungal suspension prepared by scraping spores from 5-day-old PDA slants and adding them to potato dextrose broth was incubated at 28 °C for 48 h to determine MIC. Fungal culture (100 µl) was mixed with 100 µl of different concentrations of purified compound, control blanks contained 100 µl of test compound of different concentrations and 100 µl of PDB broth, and positive control well consisted of 100 µl of fungal culture and 100 µl of PDB, negative control contained 200 µl of PDB only, and the plates were incubated at 28 °C for 48 h. Optical density was taken with a microplate reader at 595 nm. The MIC values were determined by comparing the growth in extract-containing wells to the growth in control wells, and are the lowest concentrations that resulted in a 90% inhibition of growth when compared to the control well.

### Scanning electron microscope (SEM) studies to evaluate the effect of plicacetin on cells morphology

The morphological changes in the tested bacteria (MRSA, VRE, and *B. subtills*) caused by plicacetin and standard antibiotics (vancomycin, teicoplanin, gentamicin) were investigated through SEM. The activated test bacterial turbidity was calibrated according to 0.5 McFarland standards, and an aliquot was centrifuged at 10,000×*g* for 15 min. The pellet was re-suspended in 50 µl of phosphate buffer saline (PBS) (pH 7.4) after the supernatant was discarded. This microbial cell suspension was incubated at 37 °C for 24 h after being exposed to an equal amount of plicacetin and standard antibiotics in separate vials. After incubation, the suspension was centrifuged and the final pellet of each of the treated organisms and the controls was resuspended in 30 µl PBS. SEM slides (10 mm) with thin smears were prepared and air-dried. Primary fixation was performed on the smears using a 4% (v/v) glutaraldehyde solution at room temperature for 1 h. After that, the fixed smears were given 3–5 washings with millipore distilled water, each lasting 5–10 min. These were then washed again with millipore distilled water after secondary fixation with a 4% (v/v) glutaraldehyde solution for 2 h (3–5 times). The fixed smears were then dehydrated in steps of 25%, 50%, 75%, and 95% ethanol for 10 min each, with the final dehydration stage (100% ethanol) repeated twice for 1 min each. The slides were then air-dried and placed on silver-coated SEM stubs before being examined with a scanning electron microscope (Carl Zeiss EVOLS 10 model). Positive controls included antibiotics such as vancomycin for MRSA, teicoplanin for VRE, and gentamicin for *B. subtilis*, while negative controls included untreated cells.

### Safety evaluation of Et_2_O extract and purified compound

#### Mutagenicity studies

In order to determine the mutagenicity of SP5 bioactive metabolites, an assay described by Maron and Ames^15^ was used with slight modifications recommended by Bala and Grover^16^. Salmonella Typhimurium strains TA98 and TA100 were used to investigate the antimutagenic potential of Et_2_O extract and purified compound. 4-Nitro-o-phenylenediamine (NPD, 20 μg/100 µl per plate) and sodium azide (2.5 µg/100 µl per plate) were used as a direct-acting mutagens for TA98 strain and TA100, respectively. The antimutagenicity of the compounds was determined using two methodologies:

#### Co-incubation

In the co-incubation method, top agar (2 ml) was supplemented with bacterial culture (100 μL), direct-acting mutagen (100 μL), and different concentrations of Et_2_O extract/purified compound. Afterward, minimal agar plates were incubated at 37 °C for 48 h with the top agar poured over them.

#### Pre-incubation

In the pre-incubation method, direct mutagen (100 μL) and Et_2_O ext./SP5P compound (of various concentrations) were mixed and then incubated for 30 min at 37 °C. After incubation, the mixture was added to the top agar (2 ml) along with 100 µl bacterial culture and poured onto minimal agar plates.

The number of revertant his + bacterial colonies was counted after 48 h incubation. A comparison of the number of colonies on control plates was used to determine the mutagenic potential of the compounds i.e. spontaneous (100 µL bacterial culture), positive control (100 µL bacterial culture + 100 µL mutagen), and negative control (100 µL bacterial culture + 100 µL test compound). Each concentration was tested in triplicates and each experiment was repeated twice.

The antimutagenic activity of the test compound was determined as a percent decrease in reverse mutations as follows:

$${\text{Inhibitory}}\;{\text{activity}}\;\left( \% \right) = \left[ {\left( {{\text{a}} - {\text{b}}} \right){/}\left( {{\text{a}} - {\text{c}}} \right)} \right] \times {1}00$$

a = Number of histidine revertants induced by mutagen (NPD/Sodium azide) alone, b = Number of histidine revertants induced by mutagen in the presence of test compound. c = Number of histidine revertants induced in the absence of mutagen.

#### DNA nicking assay

The method described by Lee et al.^17^ was used for DNA nicking assays. In this assay supercoiled pBR322 plasmid DNA and Fenton regent (30 mM hydrogen peroxide, 50 mM 50 mM ascorbic acid, and 80 mM FeCl_3_) were used to assess the effect of SP5 compounds to keep supercoiled pBR322 plasmid from destroying effects of hydroxyl radicals formed by Fenton’s reagent. The reaction mixture, containing plasmid DNA (1 µl), Fenton’s reagent (10 µl) and different concentrations of test compounds (10 µl), was incubated at 37 °C for 30 min. Rutin was used as a positive control. 1% agarose gel electrophoresis was done to analyze the DNA. The percentage of DNA forms i.e., supercoiled (Form I), single-stranded nicked (Form II), and linear (Form III) was calculated by densitometric analysis using Gel Quant software.

#### In vitro cytotoxicity using MTT assay

Cell culture: HEK-293T (human embryonic kidney) normal cell line was procured from National Centre for Cell Science (NCCS), Pune (India). The cell line was maintained in complete growth medium Roswell Park Memorial Institute (RPMI) 1640 supplemented with 10% fetal bovine serum along with antibiotics (100 µg/mL streptomycin and 100 Units/mL penicillin).

The cytotoxicity of the SP5P compound was examined against the HEK-293T cell line using an MTT assay following the protocol described by Mossman^43^. HEK-293T cells (5000 cells/well) were seeded in a 96-well microtiter plate and allowed to adhere overnight. Varying concentrations of SP5P compound (µg/mL) were then added to each well and further incubated for 42 h. Afterward, 100 µL of 0.5 mg/mL of MTT dye (Sigma-Aldrich) was added to each well and the plates were incubated at 37 °C in a humidified environment with 5% CO_2_ for 4 h. Then, the complete medium was aspirated from the wells and blue formazan crystals formed by the MTT reaction were dissolved in 100 µL of 100% DMSO. The color was measured at 595 nm using a microplate reader. The proliferation of cells under treatment was assessed according to the following formula:

$${\text{Cell}}\;{\text{viability}}\;\left( \% \right) = \left[ {\text{Ae/Ao}} \right] \times {1}00$$

$${\text{Growth}}\;{\text{inhibition}}\;\left( \% \right) = {1}00 - \% \;{\text{Cell}}\;{\text{viability}}$$

where Ao is the absorbance of the untreated cells (only medium) and Ae is the absorbance of the treated cells (with different concentrations of the SP5P compound).

### Supplementary Information

Supplementary Information.

## Data Availability

All data generated or analysed during this study are included in this article.

## Abbreviations

SCNA: Starch casein nitrate agar
SEM: Scanning electron microscope
MTCC: Microbial type culture collection
TLC: Thin layer chromatography
HPLC: High-performance liquid chromatography
IMTECH: Institute of Microbial Technology
