# Supplementary material for: Purification and biological analysis of antimicrobial compound produced by an endophytic Streptomyces sp
Source: Sci Rep. 2023 Sep 14;13:15248. doi: 10.1038/s41598-023-41296-x (PMC10502074; doi:10.1038/s41598-023-41296-x)
Supplement: Supplementary file 1 — Supplementary Information. [file 41598_2023_41296_MOESM1_ESM.docx]

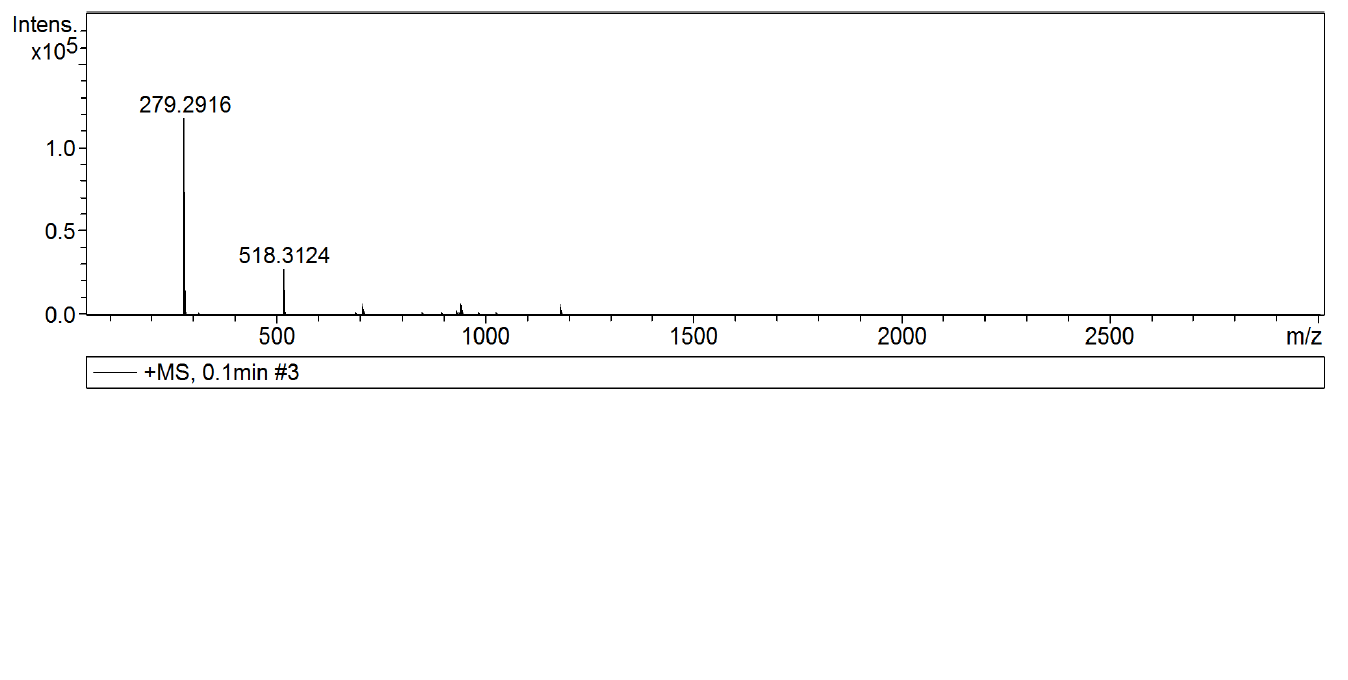


**Fig.X1: Mass spectrum of compound SP5P**

**Fig.X2: ^1^H NMR spectrum of compound SP5P**


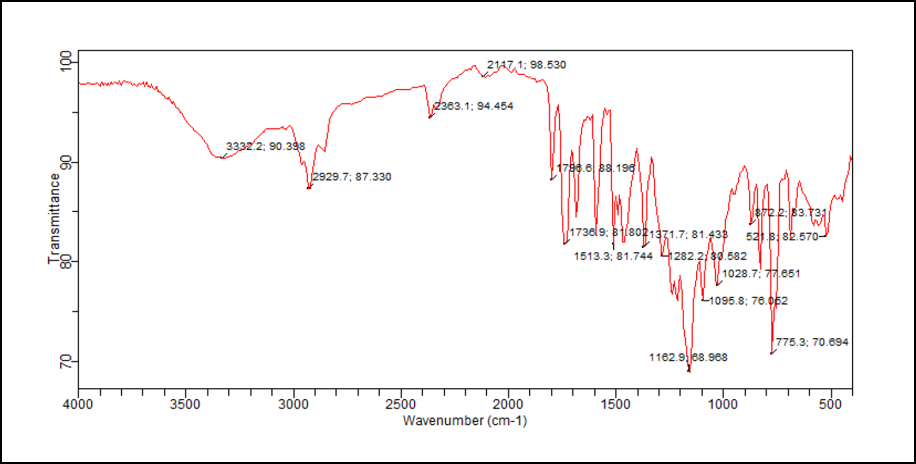


**Fig.X3: FT-IR spectrum of the compound SP5P**


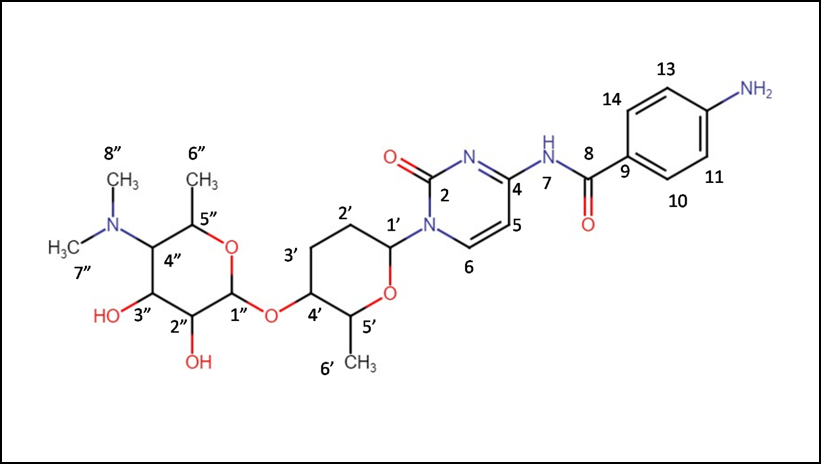


**Fig.X4: Chemical structure of purified compound SP5P**


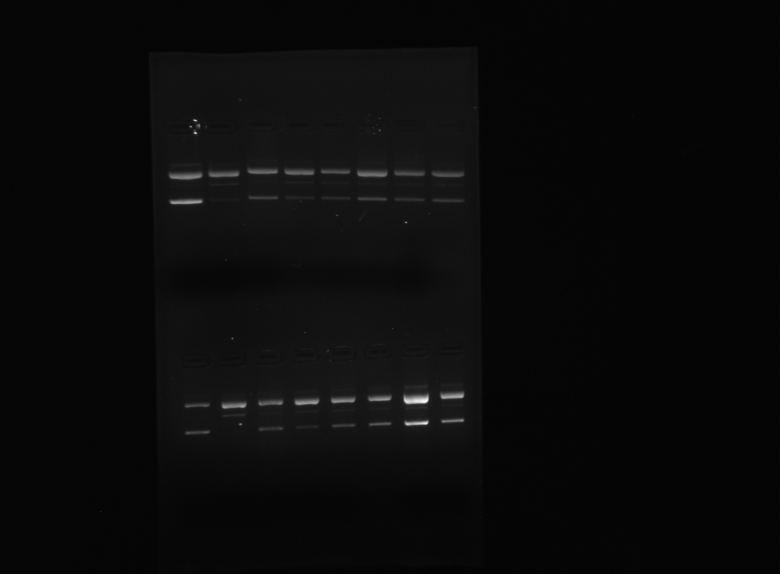


1

2

3

4

5

6

8

7

**Fig. X5**: DNA protective potential of Et_2_O extract Well 1 (negative control): only plasmid DNA; Well 2: Fenton’s reagent; Well 3: Positive control (Rutin, 10 μg); Well 4–7: Fenton’s reagent + different concentrations of Et_2_O extract (5, 10, 15 and 20μg/well); Well 8: Additional sample not related to this work


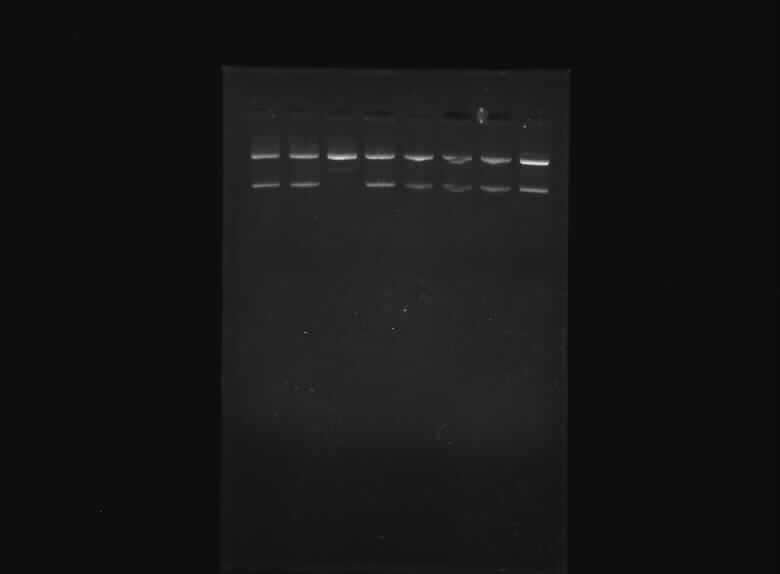


7

6

5

4

3

2

1

0

**Fig. X6:** DNA protective potential of SP5P compound purified from SP5 Well 0-1 (negative control): only plasmid DNA; Well 2: Fenton’s reagent; Well 3: Positive control (rutin, 10 μg); Well 4–7: Fenton’s reagent + different concentrations of SP5P compound (5, 10, 15 and 20μg/well)


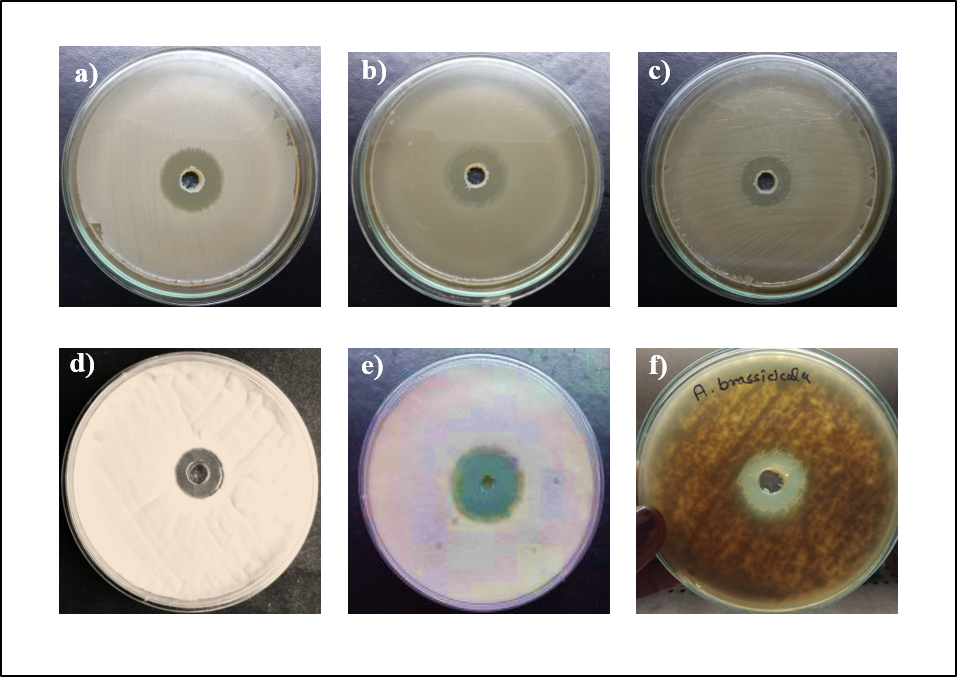


**Fig.X7:** Antimicrobial activity of Et_2_ O extract a) MRSA, b) VRE, c) *B. subtilis,* d) *F. solani*, e) *F. oxysporum*, f) *A. brassicicola*.


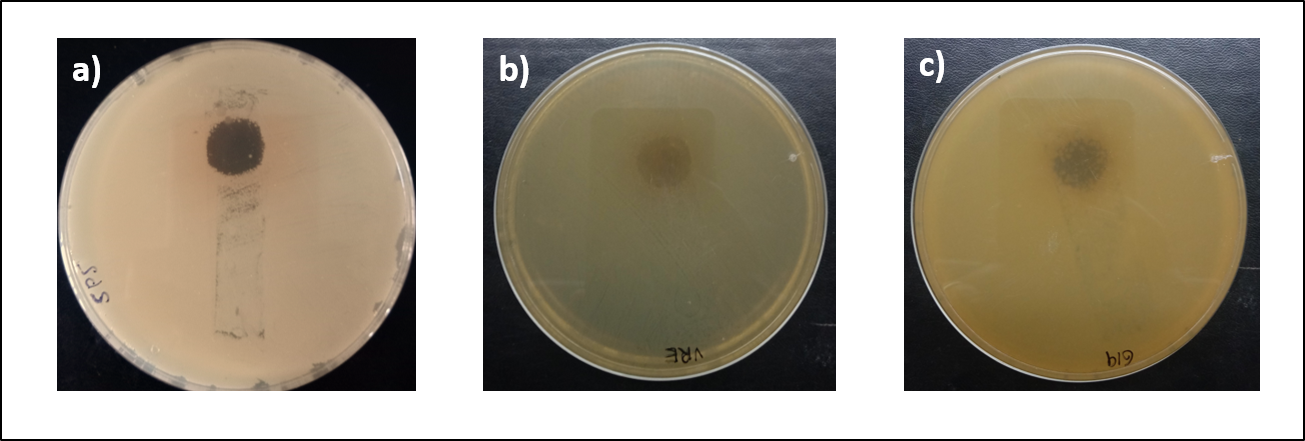


**Fig. X8:** Bioautography of *Streptomyces* sp. SP5 Et_2_ O extract against MRSA (a), VRE (b), *Bacillus subtilis* (c).

**Table: S1 Antimutagenic activity of *Streptomyces* sp. SP5 Et_2_O extract against S. Typhimurium strain TA98 and TA100**

| **Treatment** | **Concentration** | **No. of colonies** | | **Percentage inhibition (%)** | |
| --- | --- | --- | --- | --- | --- |
|  | **(µg/plate)** | **TA98** | **TA100** | **TA98** | **TA100** |
| **Spontaneous^a^** |  | 142 ± 1.4 | 354.3 ± 2.2 | - | - |
| **Positive control^b^**  **(NPD)** | 20 | 780.3 ± 1.8 |  | - | - |
| **(Sodium azide)** | 2.5 | - | 1373 ±2.5 | - | - |
| **Et_2_O extract** | 50 | 135.3 ± 1.8 | 351.6 ± 2.2 | - | - |
|  | 100 | 149 ± 1.8 | 329.3 ± 1.8 | - | - |
|  | 250 | 132 ± 1.8 | 358.6 ± 1.9 | - | - |
| **Pre-incubation** | 50 | 297.3 ± 1.1 | 671 ± 1.009 | **75.66567** | **68.91339** |
|  | 100 | 283.3 ± 1.2 | 634.6 ± 1.5 | **77.85889** | **72.48013** |
|  | 250 | 238.6 ± 1.3 | 588.6 ± 1.9 | **84.85632** | **76.99582** |
| **Co-incubation** | 50 | 359.6 ± 1.3 | 786 ± 1.2 | **65.90061** | **57.62416** |
|  | 100 | 349.3 ± 1.5 | 743.6 ± 1.7 | **67.51941** | **61.7799** |
|  | 250 | 321 ± 1.4 | 686.3 ±1.4 | **71.95808** | **67.40816** |

**Values are mean ± S.E.**

**^a^**spontaneousrevertants colonies without Et_2_O extract and mutagens

**^b^** revertants induced by mutagens

**^c^**revertants induced by Et_2_O extract

**Table.S2: Antimutagenic activity of *Streptomyces* sp. SP5 purified compound against *S.* Typhimurium strains TA98 and TA100**

| **Treatment** | **Concentration** | **No. of colonies** | | **Percentage inhibition (%)** | |
| --- | --- | --- | --- | --- | --- |
|  | **(µg/plate)** | **TA98** | **TA100** | **TA98** | **TA100** |
| **Spontaneous^a^** | - | 168.3 ± 0.6 | 232.3 ± 1.7 | - | - |
| **Positive control^b^**  **(NPD)** | 20 | 982.6 ± 1.5 | - | - | - |
| **(Sodium azide)** | 2.5 | - | 1055.3 ± 1.8 | - | - |
| **SP5P^c^** | 50 | 165.6 ± 0.9 | 231.6 ± 1.7 | - | - |
|  | 100 | 160 ± 1.2 | 227.6 ±1.6 | - | - |
|  | 250 | 162.6 ± 1.6 | 240 ± 1.4 | - | - |
| **Pre-incubation** | 50 | 300.6 ±1.3 | 354.6 ± 1.2 | **83.74901** | **85.13528** |
|  | 100 | 253.3 ±1.8 | 330.3 ± 1.03 | **89.56156** | **88.09194** |
|  | 250 | 239 ± 1.5 | 317.6 ± 0.9 | **91.3217** | **89.63102** |
| **Co-incubation** | 50 | 347 ± 1.4 | 483 ± 1.3 | **78.05926** | **69.54192** |
|  | 100 | 331.3 ± 1.5 | 460.3 ± 0.9 | **79.98314** | **72.29607** |
|  | 250 | 288.6 ± 1.1 | 418 ± 1.1 | **85.22262** | **77.43985** |

**Values are mean ± S.E.**

**^a^**spontaneousrevertants colonies without purified compound and mutagens

**^b^** revertants induced by mutagens

**^c^**revertants induced by SP5P compound

| **Treatment** | **Et_2_O** | | | **SP5P** | | |
| --- | --- | --- | --- | --- | --- | --- |
| **Form** | **I** | **II** | **III** | **I** | **II** | **III** |
| **C (Plasmid DNA)** | 44.757 | 55.242 | - | 64.72 | 46.528 | - |
| **Fenton reagent (FR)** | - | 68.466 | 31.533 | - | 67.964 | 32.035 |
| **Rutin (10µg)**  **+FR** | 31.352 | 44.174 | 24.473 | 54.328 | 45.671 | - |
| **5µg + FR** | 22.587 | 54.556 | 22.856 | 55.833 | 44.166 | - |
| **10µg + FR** | 26.891 | 49.512 | 23.596 | 57.198 | 42.801 | - |
| **15µg + FR** | 30.268 | 60.862 | 22.539 | 57.873 | 42.127 | - |
| **20 µg + FR** | 32.619 | 51.26 | 16.119 | 57.996 | 42.004 | - |

**Table.S3 DNA protective potential of Et_2_O extract and SP5P compound from *Streptomyces* sp. SP5 against free radicals generated by Fenton’s reagent**
